# Comparative Functional Genomics and the Bovine Macrophage Response to Strains of the *Mycobacterium* Genus

**DOI:** 10.3389/fimmu.2014.00536

**Published:** 2014-11-05

**Authors:** Kévin Rue-Albrecht, David A. Magee, Kate E. Killick, Nicolas C. Nalpas, Stephen V. Gordon, David E. MacHugh

**Affiliations:** ^1^Animal Genomics Laboratory, UCD School of Agriculture and Food Science, University College Dublin, Dublin, Ireland; ^2^Systems Biology Ireland, UCD Conway Institute of Biomolecular and Biomedical Research, University College Dublin, Dublin, Ireland; ^3^UCD School of Veterinary Medicine, University College Dublin, Dublin, Ireland; ^4^UCD Conway Institute of Biomolecular and Biomedical Research, University College Dublin, Dublin, Ireland

**Keywords:** cattle, BCG, gene expression, Johne’s disease, macrophage, *Mycobacterium avium* subspecies *paratuberculosis*, *Mycobacterium bovis*, tuberculosis

## Abstract

Mycobacterial infections are major causes of morbidity and mortality in cattle and are also potential zoonotic agents with implications for human health. Despite the implementation of comprehensive animal surveillance programs, many mycobacterial diseases have remained recalcitrant to eradication in several industrialized countries. Two major mycobacterial pathogens of cattle are *Mycobacterium bovis* and *Mycobacterium avium* subspecies *paratuberculosis* (MAP), the causative agents of bovine tuberculosis (BTB) and Johne’s disease (JD), respectively. BTB is a chronic, granulomatous disease of the respiratory tract that is spread via aerosol transmission, while JD is a chronic granulomatous disease of the intestines that is transmitted via the fecal-oral route. Although these diseases exhibit differential tissue tropism and distinct complex etiologies, both *M. bovis* and MAP infect, reside, and replicate in host macrophages – the key host innate immune cell that encounters mycobacterial pathogens after initial exposure and mediates the subsequent immune response. The persistence of *M. bovis* and MAP in macrophages relies on a diverse series of immunomodulatory mechanisms, including the inhibition of phagosome maturation and apoptosis, generation of cytokine-induced necrosis enabling dissemination of infection through the host, local pathology, and ultimately shedding of the pathogen. Here, we review the bovine macrophage response to infection with *M. bovis* and MAP. In particular, we describe how recent advances in functional genomics are shedding light on the host macrophage–pathogen interactions that underlie different mycobacterial diseases. To illustrate this, we present new analyses of previously published bovine macrophage transcriptomics data following *in vitro* infection with virulent *M. bovis*, the attenuated vaccine strain *M. bovis* BCG, and MAP, and discuss our findings with respect to the differing etiologies of BTB and JD.

## Introduction

*Mycobacterium* is a Gram-positive genus of Actinobacteria that includes more than 120 species (1, 2). Although the majority of species in this genus are non-pathogenic environmental bacteria, a few species are highly successful intracellular pathogens of human beings and other mammals including *Mycobacterium tuberculosis* and *Mycobacterium bovis –* the causative agents of human being and bovine tuberculosis (BTB), respectively – and *Mycobacterium avium* subspecies *paratuberculosis* (MAP), the causative agent of Johne’s disease (JD) in cattle (3, 4). The success of these pathogenic mycobacteria is partly due to their ability to infect, reside, and proliferate inside host macrophages, despite the antimicrobial properties of these cells. Macrophages serve as key effector innate immune cells that mediate the initial host response to infection via the activity of inflammatory cytokines and chemokines; this initial interaction leads to either the eradication of intracellular bacilli or the formation of organized collections of immune cells, termed granulomas, which contain infection (5).

Infections with pathogenic mycobacteria can manifest as acute or chronic disease or involve lengthy subclinical phases of infection with the potential to reactivate later. It is also understood that the establishment of successful infection is underpinned by subversion and modulation of host macrophage antimicrobial mechanisms, including the prevention of macrophage phagosome–lysosome fusion, inhibition of macrophage apoptosis, and suppression of antigen presentation and signaling mechanisms within the macrophage (6–8). Furthermore, it has been proposed that virulent mycobacteria exploit host defense mechanisms, such as the induction of cytokine-induced necrosis, which results in immunopathology, the dissemination of infection through the host and ultimately pathology that leads to shedding of the pathogen from the host, thereby maintaining the cycle of infection (9). Consequently, investigating the complex interplay between mycobacterial pathogens and the host macrophage is critical to our understanding of the immuno-pathogenesis of mycobacterial diseases.

## The *Mycobacterium tuberculosis* Complex

The genus *Mycobacterium* contains the *Mycobacterium tuberculosis* complex (MTBC) that includes seven major pathogenic species and subspecies that cause tuberculosis in a range of mammalian hosts, the most well-studied member of which is *M. tuberculosis* – the causative agent of human tuberculosis. Typically, the members of the MTBC display greater than 99.95% nucleotide sequence identity at the genome level, with little or no evidence for the exchange of genetic material between species and strains (10). Despite this high level of genome similarity, the members of the MTBC differ with respect to host range and pathogenicity: *M. tuberculosis* and *Mycobacterium africanum* are almost exclusively human pathogens; *Mycobacterium microti* causes disease in rodents including voles; *Mycobacterium pinnipedii* causes tuberculosis in marine mammals including seals and sea lions; and *Mycobacterium caprae* is very closely related to *M. bovis* and infects both goats and deer. The species with the largest host range is *M. bovis*, which is mainly isolated from cattle, but can also be responsible for outbreaks in wild animals. Furthermore, *M. bovis* can cause disease in human beings yet rarely transmits between immunocompetent hosts. A closely related mycobacterial species, *Mycobacterium canettii*, causes pathology in human beings, but differs from the other members of the MTBC in that it displays smooth colony morphology rather than the characteristic rough morphology of the other MTBC members (11, 12).

**Figure 1:**
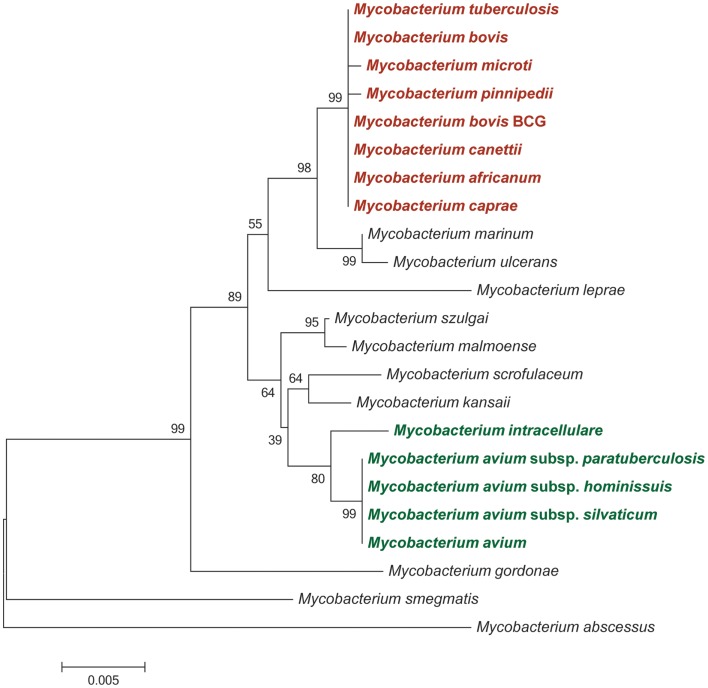
**Neighbor-joining phylogeny of selected mycobacteria species and strains based on partial 16S rRNA sequences**. Bootstrap supports are based on 1,000 pseudoreplicates. Species and strains belonging to the *Mycobacterium tuberculosis* complex are shaded in red; members of the *Mycobacterium avium* complex are shaded in green.

Phylogenetic analyses using insertion/deletion DNA sequence polymorphisms (indels), such as the variable regions of difference (RD – see below) and whole gene and genome sequences have revealed that the evolutionary history of the MTBC represents a pattern of genome downsizing characterized by chromosomal DNA sequence deletions and the inability of these species to repair deletions through recombinogenic processes (10, 13). These studies support a distinct phylogenetic position of *M. canettii* from all other MTBC members: *M. canettii* strains possess intact RD sequences that are absent from the other MTBC species together with one species-specific deletion (RD^can^). *M. canettii* strains also have 26 additional spacer sequences that are not found in other MTBC species [Figure 1] (10). Indeed, it has been recently proposed that *M. canettii* and other smooth tubercle bacilli (STB) lineages diverged from the common ancestor of all tubercle bacilli prior to the clonal radiation of non-smooth MTBC lineages and that non-smooth MTBC lineages evolved from an STB-like mycobacterial ancestor, sometimes referred to as *Mycobacterium prototuberculosis* (14).

Genomic comparisons across the MTBC revealed a number of “regions of difference” (RD loci), with the presence or absence of these loci capable of differentiating the constituent strains of the MTBC. Notably, deletion of chromosomal region RD9 distinguishes *M. tuberculosis* strains from animal-adapted lineages, including *M. bovis*, while RD1 [which encodes, among other genes, the ESX-1 secretion system plus key secreted effectors including early secretory antigen target 6 (ESAT-6) and culture filtrate protein 10 (CFP-10)] is deleted in *M. bovis* Bacille Calmette–Guérin (*M. bovis* BCG) vaccine strains. The use of deletions to differentiate mycobacterial strains led to the proposal of an evolutionary scenario positing *M. tuberculosis* as being closer to the common ancestor of the MTBC and rejection of the hypothesis that *M. tuberculosis* infection in human populations arose from an animal pathogen such as *M. bovis* in parallel with cattle domestication and husbandry (15, 16).

## The *Mycobacterium avium* Complex

A second major evolutionarily distinct cluster of mycobacterial species is represented by the *Mycobacterium avium* complex [MAC] (Figure 1). This complex shares an estimated 40% nucleotide sequence similarity with members of the MTBC based on proteome-derived DNA sequences (17). The MAC comprises several closely related, slow-growing, pathogenic, and non-pathogenic species. Among the pathogenic species within the MAC are *M. avium* and its subspecies (MAP; *M. avium* subsp. *avium*; *M. avium* subsp. *silvaticum*; and *M. avium* subsp. *hominissuis*) and *Mycobacterium intracellulare*. Phylogenetic analyses based on partial gene, whole gene, and complete genome DNA sequences have revealed that subspecies of *M. avium* typically share over ≥95% nucleotide sequence identity, while nucleotide identity between *M. avium* subspecies and *M. intracellulare* was estimated between 80 and 94% (18–21).

Despite their genetic similarity, members of the MAC show differential host and tissue tropisms. For example, *M. avium* subsp. *avium* is the classical causative agent of tuberculosis in birds, while *M. avium* subsp. *silvaticum* has been shown to cause tuberculous lesions in wood pigeons. In this regard, *M. avium* subsp. *avium* and *M. avium* subsp. *silvaticum* together represent a distinct lineage of avian pathogens. *M. avium* subsp. *hominissuis* and *M. intracellulare* are opportunistic pathogens widely distributed in the environment and can cause disseminated tuberculosis and pulmonary disease in a range of mammalian hosts, including pigs, cattle, and human beings (22). From a human perspective, *M. avium* subsp. *hominissuis* is considered to be most clinically relevant member of the MAC member, where it has been previously shown to have caused disseminated infections among immunocompromised patients (23). MAP, in contrast, is an obligate intracellular pathogen of ruminants that causes JD characterized by chronic enteritis, with severe economic losses for the dairy industry in many countries (3). MAP can be differentiated from the other subspecies of *M. avium* by its very slow growth rate *in vitro* (between 8 and 24 weeks growth is required for visible colony formation). In addition, MAP is dependent on the siderophore mycobactin J, an iron-chelating cell wall component, for growth in primary cultures (24).

## *Mycobacterium bovis* Infection and Bovine Tuberculosis

Bovine tuberculosis is caused by infection with *M. bovis*, which continues to pose a threat to livestock worldwide. Furthermore, as a zoonotic pathogen, *M. bovis* also has serious implications for human health (25). It has been estimated that BTB contributes losses of $3 billion to global agriculture annually (26, 27), while comprehensive econometric analyses place BTB as the fourth most important livestock disease worldwide (28). The impacts of BTB infection are manifold, including significant economic and social effects due to the slaughter of infected animals, compensatory payments to producers, continual surveillance programs, and disruption to agricultural trade and productivity (29).

Bovine tuberculosis is predominantly a pulmonary disease, characterized largely by the formation of tuberculous lesions in the upper respiratory lymph nodes of the lung and thorax. In some cases, tuberculous lesions have also been detected in the cranial lymph nodes (30). The etiology and host immune response to *M. bovis* is similar to *M. tuberculosis* infections in human beings (31). Infection is normally caused by the inhalation of aerosolized respiratory secretions containing infectious bacilli, with the natural site of infection being the respiratory tract, presumably on the alveolar surface of the lung. Following inhalation, the bacilli are rapidly encountered by host alveolar macrophages and other phagocytic innate immune cells (such as dendritic cells), which serve as key innate immune effector cells that provide the first line of defense against the pathogen. At this stage, bacilli can be destroyed by the antimicrobial actions of the macrophage; however, bacilli that evade intracellular destruction can persist and multiply within infected macrophages. This results in the migration of infected macrophages to regional lymph nodes, where protective T_H_1 cell immunity is induced through the recruitment and interaction of additional innate and adaptive immune cells, culminating in the formation of granulomas – organized complexes of immune cells composed of lymphocytes, non-infected macrophages, and neutrophils that contain infected macrophages and prevent the dissemination of bacilli (5, 31, 32). However, in most cases, the pathogen is not eliminated by the host; rather, the pathogen persists in a dormant stage within the granuloma for prolonged periods of time, becoming metabolically and reproductively active following the breakdown of the granuloma and dysregulation of protective T-cell immunity. This results in the development of active tuberculosis, causing immunopathology in the host and enabling the transmission of infection (31).

## MAP Infection and Johne’s Disease

Johne’s disease, caused by infection with MAP, is a chronic inflammatory disease that affects the gastrointestinal tract of cattle and other ruminants. Specifically, JD presents as a granulomatous inflammation of the intestinal tissue and regional lymph nodes due to a massive influx of monocytes and macrophages. This inflammation effectively prevents absorption of nutrients, and, therefore, during the later stages of disease cattle manifest significant weight loss and diarrhea, resulting in progressive physiological wasting and death. Disease progression is generally classified into four stages: silent infection, subclinical, clinical, and advanced clinical disease; in particular, the subclinical phase can be extremely lengthy (between 2 and 5 years), with pathology largely restricted to the ileum, rendering diagnosis difficult (24).

Exposure to MAP in ruminants generally occurs within the first months of life, through either a fecal–oral route or by ingestion of contaminated colostrum or milk, although evidence suggests that some infections can occur *in utero* (33). Following ingestion, the bacilli colonize the mucosa-associated lymphoid tissues of the upper gastrointestinal tract and are subsequently endocytosed by the microfold cells (M-cells) that cover the ileal Peyer’s patches. The bacilli are subsequently phagocytosed by subepithelial and intraepithelial intestinal macrophages, where they reside and multiply (34). The subsequent host cellular immune response leads to the development of granulomas involving adjacent lymph nodes. After years of latent infection, bacilli are assumed to reactivate and trigger a state of active cellular proliferation, leading to the corrugated intestinal epithelia and clinical manifestations with shedding of bacilli into the environment and grassland completing the infection cycle. The disease finally presents as a malnutrition syndrome that culminates in the death of the animal (3, 35, 36).

Johne’s disease has major implications for domestic animal health worldwide causing significant economic loss in affected herds, which is largely due to decreased milk yields, reduced slaughter weight, premature culling of infected animals, and losses due to continued spread of infection (37). In cattle, JD results in estimated losses of $250 million to the US dairy industry annually, while dairy herd prevalence of JD is estimated to be greater than 50% in certain US states and European countries (35, 38, 39). Furthermore, it has been hypothesized that MAP infection may trigger or exacerbate Crohn’s disease, an inflammatory disease of the intestines in human beings with similar granulomatous pathology at the ileocecal valve; however, this proposed link between MAP infection and Crohn’s disease remains contentious (40).

## The Role of the Macrophage during Mycobacterial Infection

Although BTB and JD exhibit distinct complex etiologies, the causative agents of these diseases display a propensity to infect, reside, and replicate in host macrophages – the key host innate immune cell that mediates the immune response following infection. Macrophage recognition of mycobacteria occurs through the interaction of mycobacterial pathogen-associated molecular patterns (PAMPs) – such as lipopolysaccharide, and various lipoproteins and glycolipids (e.g., lipoarabinomannan) – with host pathogen recognition receptors (PRRs) proteins, such as Toll-like receptors (TLRs), which are expressed on the macrophage cell surface (41). Macrophage PRR activation induces signaling pathways resulting in the production of endogenous NF-κB-inducible cytokines and chemokines that promote a T_H_1 immune response characterized by the release of proinflammatory IFN-γ, primarily from CD4^+^ T-cells, and the lysing of infected macrophages by cytotoxic CD8^+^ T-cells. IFN-γ induces microbicidal activity in infected macrophages and enhances the expression of major histocompatibility complex (MHC) class I and II molecules necessary for the presentation of mycobacterial antigens on the macrophage cell surface to CD8^+^ and CD4^+^ T-cells, respectively. These mechanisms can lead to either the immediate killing of the pathogen and clearing of infection, or the containment of infection through the formation of granulomas (42–45).

Pathogenic mycobacteria have evolved a diverse range of immunoevasive mechanisms that facilitate survival and replication within the host macrophage. These immunoevasive mechanisms include inhibition of phagosome maturation necessary for destruction of the pathogen and antigen presentation (46, 47); evasion of macrophage apoptosis and activation of macrophage necrosis, which facilitates release of bacilli from the macrophage and encourages dissemination of infection to other cells (7, 48); and the subversion of innate cell signaling, which is critical to the establishment of infection and progression to active disease (49, 50). It has also been recently demonstrated that virulent *M. tuberculosis* strains preferentially infect permissive macrophages and evade microbicidal macrophages through the masking of PAMPs with cell surface associated lipids (51).

Failure or subversion of an appropriate innate immune response is critical to the establishment of infection and progression to disease; central to this process is the macrophage response to infection (31). Consequently, analysis of the bovine macrophage response to *in vitro* infections with *M. bovis* and MAP may provide insights into the cellular mechanisms that underlie and govern the divergent immunopathology of BTB and JD (36, 52).

## Functional Genomics Analysis of the Bovine Macrophage Response to Mycobacteria

Early investigations of the bovine macrophage response to mycobacterial infection focused on the analysis of the expression of single or small numbers of immunological parameters. For example, the quantification of gene or protein expression using reverse transcription quantitative real-time PCR (RT-qPCR) and ELISA technologies; however, focused studies such as these, are unable to provide a high-resolution overview of the global macrophage response to infection. Pathogen-induced activation of host macrophages is characterized by large-scale changes in the expression profile of genes critical for the control and eradication of the pathogen, while modulation of host gene expression critical for pathogen survival is also expected to be reflected in the transcriptome of the macrophage (53, 54). Consequently, genomics technologies that assay pan-genomic changes in gene expression have been widely used to discern patterns of host-gene regulation during infection. In particular, the development of high-throughput gene expression technologies, such as microarrays and RNA-sequencing (RNA-seq), over the past decade, coupled with dramatic improvements in mammalian genome resources and increasingly sophisticated computational tools for the analysis of large-scale gene expression datasets are providing new opportunities for detection, cataloging, and analysis of the large numbers of host macrophage genes expressed in response to mycobacterial infection in cattle (55–64).

A primary goal of our research group is to use high-throughput functional genomics technologies to analyze the bovine macrophage transcriptome following infection with *M. bovis* and *M. paratuberculosis* to improve our understanding of host–pathogen interactions that characterize and underlie BTB and JD. Previously, we used a 24-h time course infection model to investigate the transcriptome response of bovine monocyte-derived macrophages (MDM) infected with *M. bovis* and MAP using data generated from the Affymetrix^®^ GeneChip^®^ Bovine Genome microarray platform (61, 62). These analyses revealed a large number of differentially expressed (DE) genes following *M. bovis* infection relative to non-infected control MDM such that the number of DE genes also increased across the time course, with the highest number observed 24 h post-infection [hpi] (62). This contrasted with results from MAP-infected MDM relative to the same control macrophages, which showed the highest number of DE genes at the 2 hpi time point with a decrease in the number of DE genes at the later time points post-infection (i.e., 6 and 24 hpi) (61). These findings suggest that *M. bovis* and MAP have differential survival strategies once internalized by macrophages, which in turn, may underlie the divergent immunopathology associated with BTB and JD.

## The Monocyte-Derived Macrophage Infection Model and Gene Expression Datasets Used for Comparative Functional Analysis

To further investigate the similarities and differences of the bovine MDM response to virulent and attenuated mycobacterial species/strains, we have reanalyzed and directly compared the Affymetrix^®^ GeneChip^®^ Bovine Genome microarray data from our earlier work (i.e., the non-infected control MDM and the *M. bovis*- and MAP-infected bovine MDM) together with corresponding microarray data from MDM infected with *M. bovis* BCG (65). All infected and control MDM used to generate these data were derived from the same seven age-matched Holstein-Friesian females, while a multiplicity of infection (MOI) of 2:1 (i.e., 2 bacilli:1 MDM) was used for all MDM infections (61, 62). Gene expression omnibus (GEO) data series accession numbers used for these re-analyses were GSE33309, GSE35185, and GSE59774 (66).

**Figure 2:**
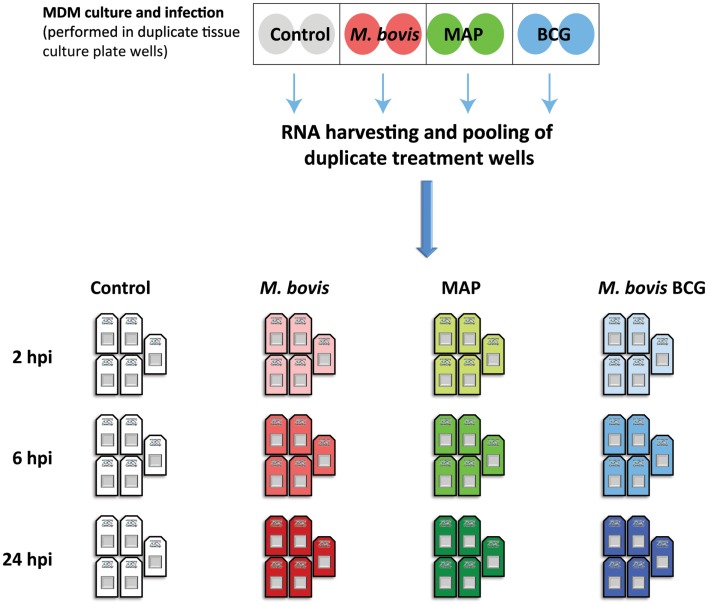
**The experimental design used for the comparative functional genomics analysis described in the current study**. Previously, MDM from seven age-matched females were infected with *M. bovis* and MAP (MOI 2:1); control MDM received culture media only (61, 62). We also infected, in parallel, MDM from the same animals with *M. bovis* BCG (MOI 2:1). Infections were performed across duplicate tissue culture plate wells (shaded circles); total RNA from duplicate treatment wells was harvested and pooled at 2, 6, and 24 hpi. Pan-genomic gene expression data for each RNA sample was generated using the Affymetrix^®^ GeneChip^®^ Bovine Genome microarray platform (61, 62). For the comparative functional genomics analysis, microarray data from only five of these animals were used to ensure a balanced experimental design following quality control assessment (see main body text for details).

For this new comparative analysis, gene expression data from *M. bovis*-, MAP-, and *M. bovis* BCG-infected MDM together with data from the non-infected control MDM at time points 2, 6, and 24 hpi were used. Prior to differential gene expression analysis, all microarray data were quality checked using the arrayQualityMetrics package in Bioconductor (67). Raw data from two microarrays (one MAP- and one *M. bovis* BCG infected MDM sample) did not pass the QC thresholds set in the arrayQualityMetrics package and were removed from all further downstream analyses. Furthermore, all arrays generated from these two animals were excluded to ensure a balanced experimental design for comparative gene expression analysis (Figure 2). Next, all raw gene expression data were normalized and filtered for non-informative probe sets using the I/NI algorithm implemented in the FARMS Bioconductor software package (version 1.14.0) (68); this analysis yielded 11,842 informative probe sets for use in downstream analysis.

**Figure 3:**
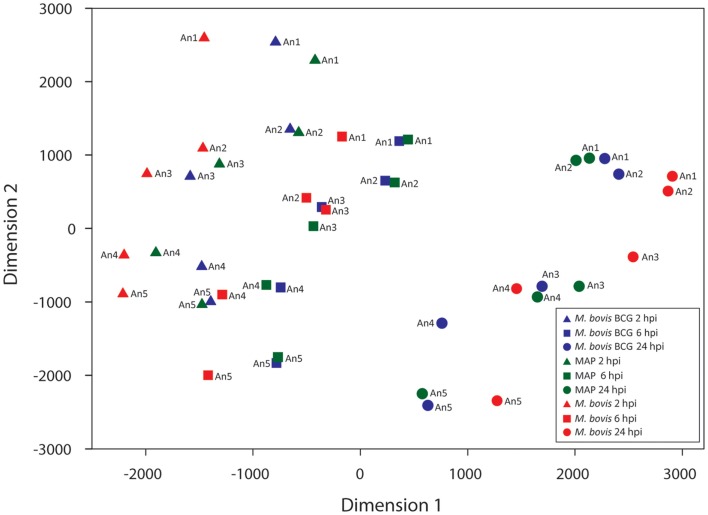
**Multi-dimensional scaling (MDS) plot of the infected MDM at each time point post-infection**. Manhattan distances (calculated from 11,842 informative probe sets) were used to generate the MDS plot.

The 11,842 informative probe sets identified post-filtering were then used to generate a multi-dimensional scaling (MDS) plot summarizing the transcriptomic relationship between samples (Figure 3). Notably, samples clustered according to time on the first dimension, while the second dimension clustered samples according to animal ID. However, there was a noticeable clustering of RNA samples from *M. bovis* BCG- and MAP-infected samples within each group of samples corresponding to a particular animal at a given time. Investigation of the expressed genes with the greatest animal effect revealed that loci within the bovine MHC displayed the greatest differences in expression among animals with relatively small gene expression differences within each animal across time and treatment (Figure S1 in Supplementary Material). The magnitude of the expression differences among animals for these genes presumably contributes to the separation of the samples by animal on the second dimension in the MDS plot (Figure 3).

We propose two hypotheses to explain the observed expression differences among animals. First, expressed MHC loci, which are among the most polymorphic loci in mammals, generate mRNA transcripts that display considerable nucleotide differences between individuals from the same species (69–71). mRNA transcripts that vary appreciably from the reference transcript sequences used to produce the microarray probe sets may, therefore, have a reduced hybridization efficiency compared to mRNA transcripts that are identical to or differ only slightly from the reference transcript sequences. In turn, this may result in Type 1 errors for differential gene expression estimates between samples or sample groups (72–74). Consequently, the animal effect observed in these data may be due, in part, to the technical limitations of the microarray platform used. Second, it is possible that the observed inter-animal differential gene expression is due, in part, to real differences in mRNA abundance generated by genotypic differences at loci that regulate gene expression (71, 74, 75); indeed, genotypic differences at loci that regulate gene expression in response to mycobacterial infection may contribute to phenotypic differences in the ability of an animal to clear or succumb to infection. In addition, a combination of both these technical and biological factors could also explain the observed animal effect.

## Comparative Functional Genomics Analysis Reveals Similarities and Differences in the Macrophage Transcriptome Response to *M. bovis*, MAP, and *M. bovis* BCG

**Figure 4:**
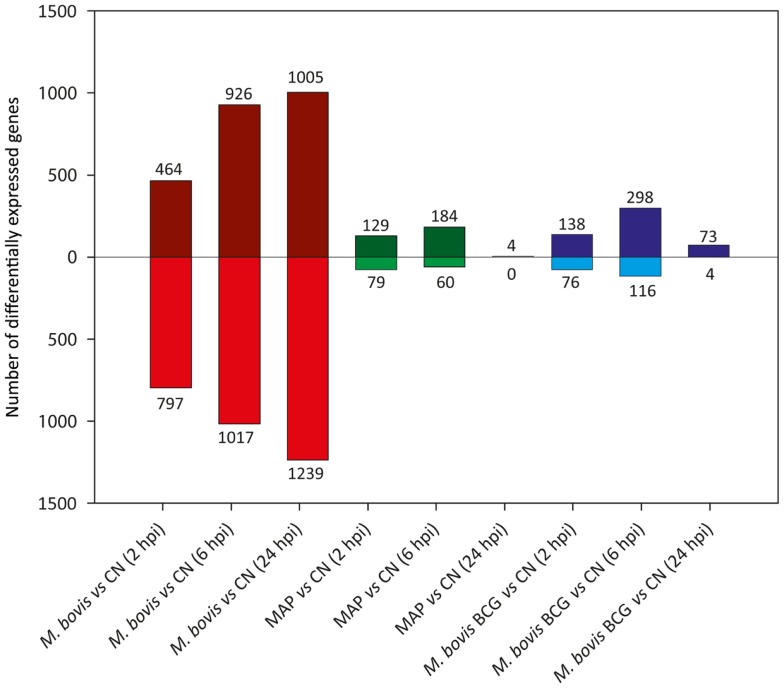
**The number of DE genes found for paired comparisons of infected MDM (i.e., MDM infected with *M. bovis*/MAP/*M. bovis* BCG) relative to the control MDM for each time point post-infection (FDR ≤ 0.05)**. Bars above the horizontal line (i.e., *y* = 0) indicate the number of genes displaying upregulation in the infected MDM relative to the control MDM; bars below the horizontal line indicate the number of genes displaying downregulation in the infected MDM relative to the control MDM.

Figure 4 shows the results of differential gene expression analysis for the infected MDM (i.e., *M. bovis*, MAP, and *M. bovis* BCG) relative to the non-infected control MDM [false-discovery rate (FDR) adjusted *P* value ≤0.05]. Notably, for each infected MDM/control, MDM contrast the number of DE genes varied with respect to time. *M. bovis*-infected MDM exhibited the greatest number of DE genes, with the number of DE genes increasing across the 24-h time course. Furthermore, for *M. bovis*, the number of downregulated genes exceeded the number of upregulated genes at each of the time points post-infection.

In contrast, the number of DE genes observed for both the MAP- and *M. bovis* BCG-infected MDM (relative to the non-infected controls) was highest at the 6 hpi time point for both sample groups (Figure 4). These results indicate that for MAP and *M. bovis* BCG infection, MDM differential gene expression had largely abated at the 24 hpi time point and that the MDM transcriptome reverted to a transcriptional state similar to that of the control MDM. These observations support previous work that showed that differential gene expression changes in MAP-infected bovine MDM are transient and are largely undetected 24 hpi relative to non-infected control MDM (76, 77).

**Figure 5:**
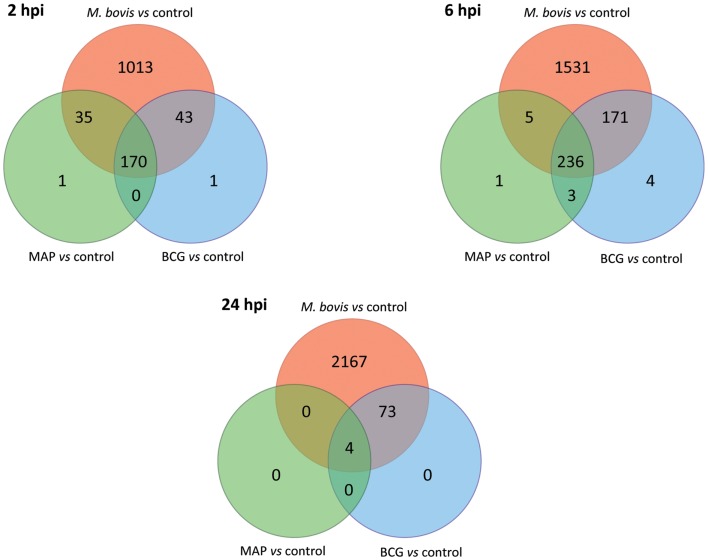
**The number of shared and unique DE genes among the three infected MDM groups based on gene expression data relative to the control MDM (FDR ≤ 0.05)**. Red shading denotes *M. bovis*-infected MDM; green shading denotes MAP-infected MDM; blue shading denotes *M. bovis* BCG (BCG)-infected MDM.

A comparison of the lists of DE genes obtained for all mycobacteria/control contrasts at each time point (Figure 5) identified a core set of DE genes at the 2 and 6 hpi time points common to all three mycobacterial treatments consisting of 170 and 236 DE genes, respectively. Among the DE genes common to all three mycobacterial infections were *IL1A*, *IL1B*, *TNF*, *NFKB1*, and *NFKB2*; all of these genes were upregulated at one or more time points in all types of infected MDM, suggesting a robust inflammatory reaction to all of the mycobacteria used in this study. Ingenuity^®^ Systems Pathway Analysis (IPA; Ingenuity Systems, Redwood City, CA, USA; www.ingenuity.com) was used to identify canonical pathways within the list of DE genes that were common to all three mycobacterial treatments relative to the control group. The top ranking canonical cellular pathways (based on the lowest adjusted *P*-values; FDR ≤ 0.05) enriched for the 170 and 236 common DE genes identified at 2 and 6 hpi included *IL-10 signaling* (2 hpi); *dendritic cell maturation* (2 and 6 hpi); *IL-6 signaling* (2 hpi); *TNFR2 signaling* (2 hpi), *TWEAK signaling* (2 hpi); *communication between innate and adaptive immune cells* (6 hpi), *NF-κB signaling* (6 hpi); and *TREM1 signaling* (2 and 6 hpi; FDR ≤ 0.0001) (see Tables S1 and S2 in Supplementary Material).

We also observed large numbers of DE genes that were specific to *M. bovis*-infected MDM, which increased over time from 1,013 significant genes at 2 hpi to 2,167 at 24 hpi (Figure 5). The percentages of DE genes unique to *M. bovis* infection relative to the total number of DE genes detected following *M. bovis* infection were 80.3% (2 hpi), 78.8% (6 hpi), and 96.6% (24 hpi). IPA analysis of the DE genes unique to *M. bovis*-infected macrophages across all three time points (Tables S3–S5 in Supplementary Material) revealed enrichment for genes involved in cell signaling, including *IL-6 signaling* and *mitogen-activated protein kinase (MAPK) signaling*, the latter of which regulates the expression of several transcription factors, such as those encoded by *FOS* and *JUN* that are critical for the activation of immune cells (78). In contrast, the number of DE genes specific to MAP and *M. bovis* BCG infection across the time course was markedly lower; for example, no gene was specific to either MAP or *M. bovis* BCG infection 24 hpi. Indeed, for each post-infection time point, more than 98.3% of the DE genes induced by MAP and *M. bovis* BCG relative to the controls were among the list of DE genes induced by *M. bovis* relative to the controls.

**Figure 6:**
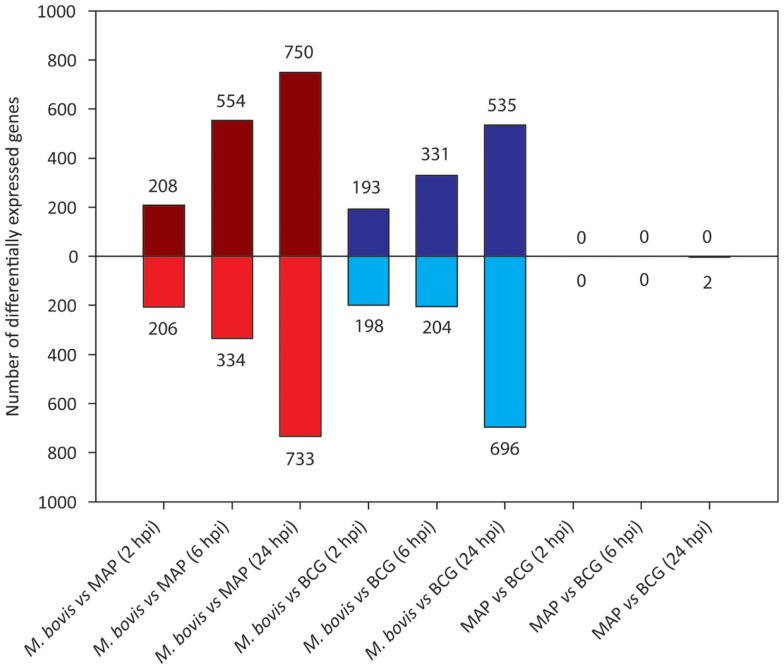
**The number of DE genes found for pairwise comparisons among infected MDM groups (FDR ≤ 0.05)**. Bars above the horizontal line (i.e., *y* = 0) indicate the number of genes displaying upregulation; bars below the horizontal line indicate the number of genes displaying downregulation.

We next analyzed differential gene expression directly between each pair of mycobacteria-infected sample groups (Figure 6). Large and increasing numbers of DE genes were found across the time course in *M. bovis*-infected MDM relative both MAP- and BCG-infected MDM, confirming the divergence between the two types of MDM transcriptional responses. Notably, no DE genes were detected between MAP- and BCG-infected MDM at 2 and 6 hpi, while only two DE genes were identified at 24 hpi; this contrasts with the larger numbers of DE genes identified through the indirect comparison of MAP- and BCG-infected MDM using the controls as a common reference (Figure 5). This discrepancy is most readily explained by differences in variances of gene expression within each sample group as illustrated in Figure S2 in Supplementary Material.

The overlap in DE genes across all three treatment groups (relative to the control groups) at the 2 and 6 hpi time points suggest that a “core” MDM transcriptional response is induced by all three mycobacterial species/strains during the early stages of infection. This core transcriptome is characterized by genes involved in innate cytokine signaling and production, which encode proteins that activate the adaptive immune response following mycobacterial infection. However, the large and increasing number of DE genes specific to *M. bovis* across the infection time course demonstrates that *M. bovis* is a more potent inducer of proinflammatory genes than *M. bovis* BCG or MAP and highlights the distinct MDM gene expression profile elicited in response to this pathogen. In addition, the enrichment of *M. bovis*-specific DE genes for roles in macrophage cell signaling, such as IL-6 and MAPK signaling, suggests that additional cellular pathways are triggered by this pathogen relative to *M. bovis* BCG or MAP.

Although proinflammatory cytokines and chemokines play a pivotal role in mediating the host immune response to control mycobacterial infection, several lines of evidence suggest that these molecules and their associated pathways can be exploited by virulent mycobacterial pathogens to promote granuloma formation, which recruit new macrophages to the site of infection enabling persistence within the host (79). For example, non-regulated production of proinflammatory cytokines and chemokines can result in immunopathology, including destructive inflammation and necrosis, allowing dissemination of the pathogen from infected cells (9, 80, 81). Therefore, the immunopathology of BTB may be associated with the increased induction of innate immune genes following infection with *M. bovis*. Furthermore, the divergent transcriptomic profile observed in MDM infected with virulent *M. bovis* relative to *M. bovis* BCG-infected MDM is presumably governed, in part, by the presence of several secreted virulence factors, such as those encoded by the RD1 locus (57, 82, 83). This locus is present in all virulent strains of *M. bovis* (and *M. tuberculosis*) but is absent in attenuated strains of *M. bovis* BCG (84).

Conversely, the reduced number of DE genes detected between the MAP-infected and the non-infected control and *M. bovis* BCG-infected MDM across the time course suggests that MAP infection does not result in a major perturbation of the MDM transcriptome; rather, bovine MDM sense and respond to MAP in a similar manner to attenuated *M. bovis* BCG despite their markedly distinct evolutionary histories and different pathogenicities. These results support the hypothesis that MAP infection of host MDM is achieved via a capacity to appear “benign,” which enables it to reside and replicate within the macrophage for prolonged periods of time, and may underlie the lengthy subclinical phase of infection characteristic of JD (85). Our results also suggest that the immunoevasive mechanisms used by MAP involve the suppression of the proinflammatory response, such that the transcriptome of an infected macrophage resembles that of a non-infected cell. In support of this, *in vivo* MAP infection models demonstrate that cattle initially develop an early proinflammatory and T_H_1-type response to infection, which gradually declines in animals that progress to active disease, favoring a T_H_2-type response that does not control infection (3, 86–88). Notably, the immunosuppression observed *in vivo* may originate at a cellular level, whereby MAP-infected macrophages fail to properly respond to host-derived immune activators such as CD40L and IFN-γ (89, 90).

## Transcriptional Evidence for Type I Interferon-Mediated Regulation of Interleukin I Production in *M. bovis*-Infected MDM: Toward a Mechanism of Pathology

Examination of the lists of DE genes for each mycobacterial infection/control contrast shows that type I interferon-inducible genes such as *IFIT1*, *IFIT2*, *MX1*, *MX2*, and *IL27* and interferon-dependent *CXCL10* were not DE following MAP and *M. bovis* BCG infection at any of the post-infection time points. However, all of these genes were DE for at least one time point following *M. bovis* infection (Table 1). Similarly, the gene encoding type II interferon (i.e., *IFNG*) was also upregulated at 6 and 24 hpi following *M. bovis* infection, but was not DE at any post-infection time point following infection with MAP and *M. bovis* BCG. We further observed that, in general, the fold-change of upregulation of the type I and type II interferon-inducible genes increased over the time course of infection in the *M. bovis*-infected MDM, with an accompanying decrease in the fold-change of upregulation of the interleukin-1 genes (*IL1A* and *IL1B*).

**Table 1:** **Log_2_ fold-change values of manually curated genes for several immune-related pathways**.

	*M. bovis* vs CN			*M. bovis* BCG vs CN			MAP vs CN			*M. bovis* vs *M. bovis* BCG			*M. bovis* vs MAP		
	2 hpi	6 hpi	24 hpi	2 hpi	6 hpi	24 hpi	2 hpi	6 hpi	24 hpi	2 hpi	6 hpi	24 hpi	2 hpi	6 hpi	24 hpi
**IL-1 signaling genes**
*IL1B*	5.68	4.04	2.85	4.14			3.77			1.54	2.80	1.80	1.91	2.71	2.18
*IL1A*	5.37	2.42	2.50	4.21			3.88			1.16	1.91	1.64	1.49	1.85	2.16
**Type 1 interferon-related genes**
*IFNB1*			0.76									0.79			0.66
*IFNAC*			0.17									0.18			0.18
*CXCL10*	2.04	2.89	3.00											2.10	1.92
*IFIT2*			2.27									1.94			1.70
*MX1*			1.18									1.07			0.85
*MX2*			1.99									1.43			1.41
*IFNAR1*					0.33										
*IFNAR2*	0.20	0.24	0.25	0.20	0.21		0.19	0.17				0.20			0.25
**Type 2 interferon-related genes**
*IFNG*		2.06	1.88								1.49			1.78	2.02
*IFNGR2*															
*IFNGR2*			0.15												
**NF-κB-related genes**
*NFKB1*	1.47	1.19	0.61	0.98	0.89		1.00	0.58		0.50		0.43	0.48	0.61	0.53
*NFKB2*	2.59	3.07	1.84	1.74	2.30		1.69	1.87		0.85	0.77	1.14	0.89	1.20	1.38
*TNF*	5.11	3.98	2.30	4.11	1.39		3.55	1.47			2.59	1.52	1.57	2.51	2.04
*IL6*	4.49	2.15	3.12	2.86			2.40			1.63	1.67	2.20	2.09	1.57	2.73

*Cells containing values are statistically different for the contrasts listed in the column headings (FDR ≤ 0.05); empty cells are not statistically different (FDR ≥ 0.05). The intensity of the shading is related to relative increased fold-changes in gene expression*.

IL-1 and type I IFN-signaling pathways have been shown to play important, yet opposing, roles in determining the host response to infection with virulent members of the MTBC. Mice deficient in IL-1B display increased susceptibility to virulent *M. tuberculosis*, indicating that IL-1 signaling is required for the host control of infection (91, 92). Conversely, mice deficient in type I IFN signaling show reduced bacterial loads following infection, suggesting that type I IFN plays a contributory role in tuberculosis disease progression (93, 94). Studies have also shown that virulent *M. tuberculosis* and attenuated *M. bovis* BCG use distinct signaling pathways for regulating IL-1B production in human MDM. *M. tuberculosis* induced the expression of IFN-related genes, while induction of type I IFN-signaling inhibited IL-1B secretion (95, 96). Notably, infection of human MDM with *M. bovis* BCG did not induce significant differential expression of IFN-related genes or IL-1B secretion. These results suggest that type I IFN-mediated suppression of IL-1B production is a key mechanism for the intracellular survival of *M. tuberculosis* (95, 96). Comparable *in vivo* and *in vitro* studies of bovine ileal tissue and MDM have demonstrated increased transcript and protein levels of IL-1A and IL-1B (*in vitro* only) in response to MAP infections, with a concomitant increase in downstream expression of TRAF1 (97, 98). This increase in TRAF1 has been proposed to enhance survival of MAP in macrophages due to the anti-apoptotic properties of TRAF1 and its capacity to interfere with normal macrophage activation, particularly via CD40/CD40L (98, 99).

**Figure 7:**
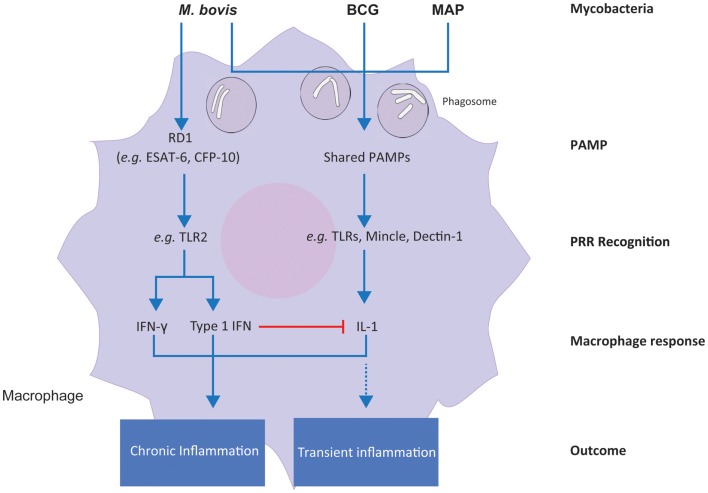
**A proposed model for the differential responses of bovine MDM to *M. bovis*, *M. bovis* BCG, and *M. avium* subsp. *paratuberculosis* infection *in vitro***. Differential integration of common and specific signals induced by the three mycobacterial types is shown.

The increase in type I IFN gene expression and the concomitant decrease in *IL1* induction that we observed in *M. bovis*-infected MDM over the 24-h infection time course suggests that the intracellular survival strategy of virulent *M. bovis* may also involve type I IFN-mediated suppression of *IL1* production. In contrast to this, these results suggest that the same immunoevasive mechanism is not used by virulent MAP or the attenuated *M. bovis* BCG in bovine MDM. These results indicate that differential activation of macrophage immunoregulatory pathways is central to the differential intracellular survival mechanisms of these related, yet distinct, bovine mycobacterial pathogens. A proposed model of the differential response of bovine MDM to the mycobacteria examined in this comparative study is shown in Figure 7.

## Differential Mycobacterial Virulence Factors and Their Impact on Macrophage Pathogen Recognition

The region of difference 1 (RD1) locus, which is present in *M. bovis*, is a major genetic difference between this species and MAP and *M. bovis* BCG, which both lack RD1 (100). Moreover, RD1 (which is also present in virulent *M. tuberculosis* strains) has attracted increasing interest over the last two decades, because it contains the ESX-1 type VII secretion system, responsible for the secretion of virulence factors, such as the dimer ESAT-6/CFP-10 (101). ESAT-6, which is proposed to facilitate escape from macrophage phagolysosomal degradation, binds to TLR2 receptors, and activates TLR signaling cascades within the macrophage that culminate in cytokine production (102–104). It has been recently reported that TLR2 receptors mediate enhanced interferon production through reprograming of murine macrophages following infection with viral ligands (105). In support of these observations, our results showed that differential expression of both type I and type II interferon genes is unique to *M. bovis*-infected MDM, which is not present in MDM infected with MAP and *M. bovis* BCG. Consequently, we hypothesize that the virulence factors encoded in the RD1 region and secreted by *M. bovis* – but not MAP or *M. bovis* BCG – trigger an additional cascade of signaling events, such as those mediated by TLRs and MAPKs (as revealed through IPA analyses of unique *M. bovis*-induced genes), relative to attenuated *M. bovis* BCG and the lengthy subclinical MAP. In turn, the combined activation of additional immune pathways and canonical PRR-dependent pathways may lead to a sustained (i.e., chronic) inflammatory response in infected macrophages, as opposed to a more transient inflammation following *M. bovis* BCG or MAP infections.

## Concluding Remarks

In the present study, we highlight markedly different transcriptional response of bovine MDM infected with *M. bovis* over a 24-h time course compared to the closely related but attenuated *M. bovis* BCG strain and to virulent MAP. We hypothesize that RD1-encoded virulence factors provide a mechanistic basis for this differential response, as RD1 was lost during the derivation of the *M. bovis* BCG vaccine strain from *M. bovis* and is absent from the MAP genome. We also identified a common MDM transcriptional response to both attenuated *M. bovis* BCG and MAP. We propose that the respective attenuated and lengthy subclinical phenotypes of *M. bovis* BCG and MAP may induce similar responses in infected macrophages, at least during the early stages of infection. Finally, we identified type I interferon-dependent genes among the DE genes specific to virulent *M. bovis-*infected MDM, adding further evidence supporting a key role for type I interferon in the establishment of active tuberculosis in cattle as has previously reported for human tuberculosis (106).

While the comparative functional genomics analysis presented here is based on data generated from microarrays, the changing landscape of transcriptomics, as represented by the advent of high-throughput RNA-seq, offers unprecedented opportunities to study the host macrophage response to mycobacterial infection at the nucleotide level, including investigation of the effect of genotype on gene expression levels. High-throughput sequencing technologies are providing novel insights into the cellular mechanisms governing mycobacteria–macrophage interactions, enabling further understanding of how modulation of these pathways can result in pathology. In addition, identification of transcriptional biomarkers of infection may lead to the development of novel diagnostics for BTB and JD, providing new molecular tools for disease control and eradication.

## Conflict of Interest Statement

The authors declare that the research was conducted in the absence of any commercial or financial relationships that could be construed as a potential conflict of interest.The Guest Associate Editor Kieran G. Meade declares that, despite having collaborated with authors Kévin Rue-Albrecht, David A. Magee, Kate E. Killick, Nicolas C. Nalpas, Stephen V. Gordon and David E. MacHugh, the review process was handled objectively and no conflict of interest exists.

## Supplementary Material

The Supplementary Material for this article can be found online at http://www.frontiersin.org/Journal/10.3389/fimmu.2014.00536/abstract
