# Supplementary material for: Comparative Functional Genomics and the Bovine Macrophage Response to Strains of the Mycobacterium Genus
Source: Front Immunol. 2014 Nov 5;5:536. doi: 10.3389/fimmu.2014.00536 (PMC4220711; doi:10.3389/fimmu.2014.00536)
Supplement: Supplementary file 6 [file Data_Sheet_1.DOCX]

***Supplementary Material***

**Comparative functional genomics and the bovine macrophage response to strains of the *Mycobacterium* genus**

**Kévin Rue-Albrecht^1^, David A. Magee^1,†^, Kate E. Killick^1,2^, Nicolas C. Nalpas^1^, Stephen V. Gordon^3,4^, David E. MacHugh^1,4^***

^1^Animal Genomics Laboratory, UCD School of Agriculture and Food Science, University College Dublin, Belfield, Dublin 4, Ireland

^2^Systems Biology Ireland, UCD Conway Institute of Biomolecular and Biomedical Research, University College Dublin, Dublin 4, Ireland

^3^UCD School of Veterinary Medicine, University College Dublin, Belfield, Dublin 4, Ireland

^4^UCD Conway Institute of Biomolecular and Biomedical Research, University College Dublin, Belfield, Dublin 4, Ireland

*** Correspondence:** David E. MacHugh, Animal Genomics Laboratory, UCD School of Agriculture and Food Science, University College Dublin, Belfield, Dublin 4, Ireland.

[david.machugh@ucd.ie](file:///C:\Users\DMacHugh\Dropbox\Publications\RueAlbrecht_Frontiers.In.Immunology(Review)\Current%20version\david.machugh@ucd.ie)

**† Present address:** Department of Animal Science, University of Connecticut, Storrs, CT 06269-4040, USA.

**Keywords: cattle, BCG, gene expression, Johne’s disease, macrophage, *Mycobacterium avium* subspecies *paratuberculosis*, *Mycobacterium bovis*, tuberculosis**

1. **Supplementary Figures and Tables**

**Table S1:** The canonical pathways identified by IPA^®^ for the common DE genes (relative to the control group) detected at 2 hpi. [**Table_1.xls**]

**Table S2:** The canonical pathways identified by IPA^®^ for the common DE genes (relative to the control group) detected at 6 hpi. [**Table_2.xls**]

**Table S3:** The canonical pathways identified by IPA^®^ for the DE genes unique to *M. bovis*-infected MDM detected at 2 hpi. [**Table_3.xls**]

**Table S4:** The canonical pathways identified by IPA^®^ for the DE genes unique to *M. bovis*-infected MDM detected at 6 hpi. [**Table_4.xls**]

**Table S5**: The canonical pathways identified by IPA^®^ for the DE genes unique to *M. bovis*-infected MDM detected at 24 hpi. [**Table_5.xls**]

**Figure S1:** An example of probe sets mapping to bovine major histocompatibility complex (BOLA locus) genes showing a strong animal effect on gene expression. [**Image_1.tif**]

**Figure S2:** Two example probe sets showing the effect of variance in gene expression on differential expression. (A) Probe set Bt.5420.1.A1_at (gene ID PCP4L1) at 6 hpi shows differential expression for each infected MDM group relative to the control group (FDR ≤ 0.05). M. bovis-infected MDM also show differential expression relative to MAP and M. bovis-BCG-infected MDM (FDR ≤ 0.05); however, no difference in expression was observed between MAP- and M. bovis-BCG-infected MDM. (B) Probe set Bt.24741.1.S1_at (non-annotated gene) at 6 hpi displays differential expression between M. bovis-BCG-infected MDM relative to the control MDM group but not to MAP-infected MDM (FDR ≤ 0.05); while MAP-infected MDM are not differentially expressed relative to the control group (FDR ≥ 0.05). [**Image_2.tif**]
