# Supplementary figures and images for: Comparative Functional Genomics and the Bovine Macrophage Response to Strains of the Mycobacterium Genus
Source: Front Immunol. 2014 Nov 5;5:536. doi: 10.3389/fimmu.2014.00536 (PMC4220711; doi:10.3389/fimmu.2014.00536)

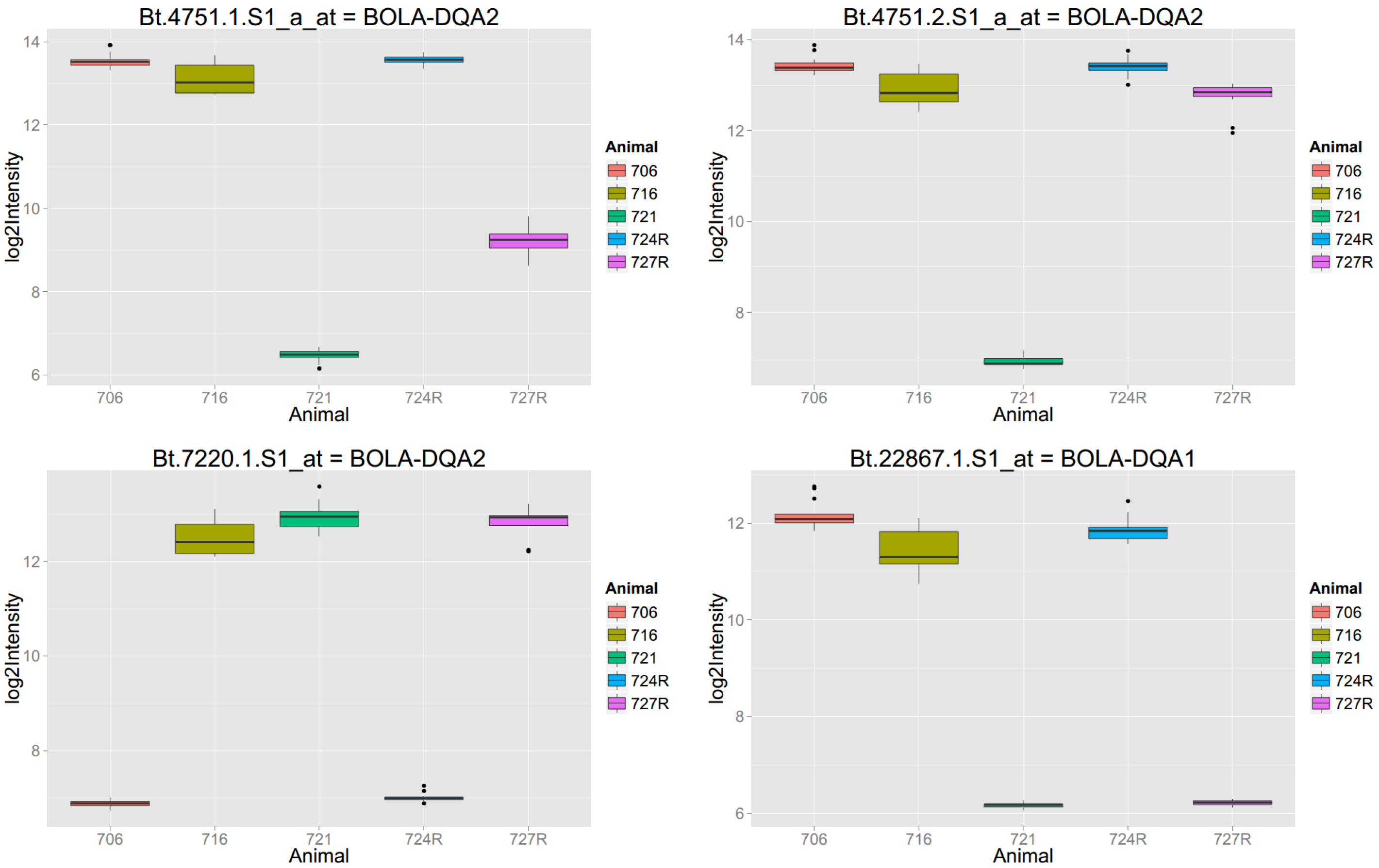

Supplement: Supplementary file 7 [file Image_1.TIF]

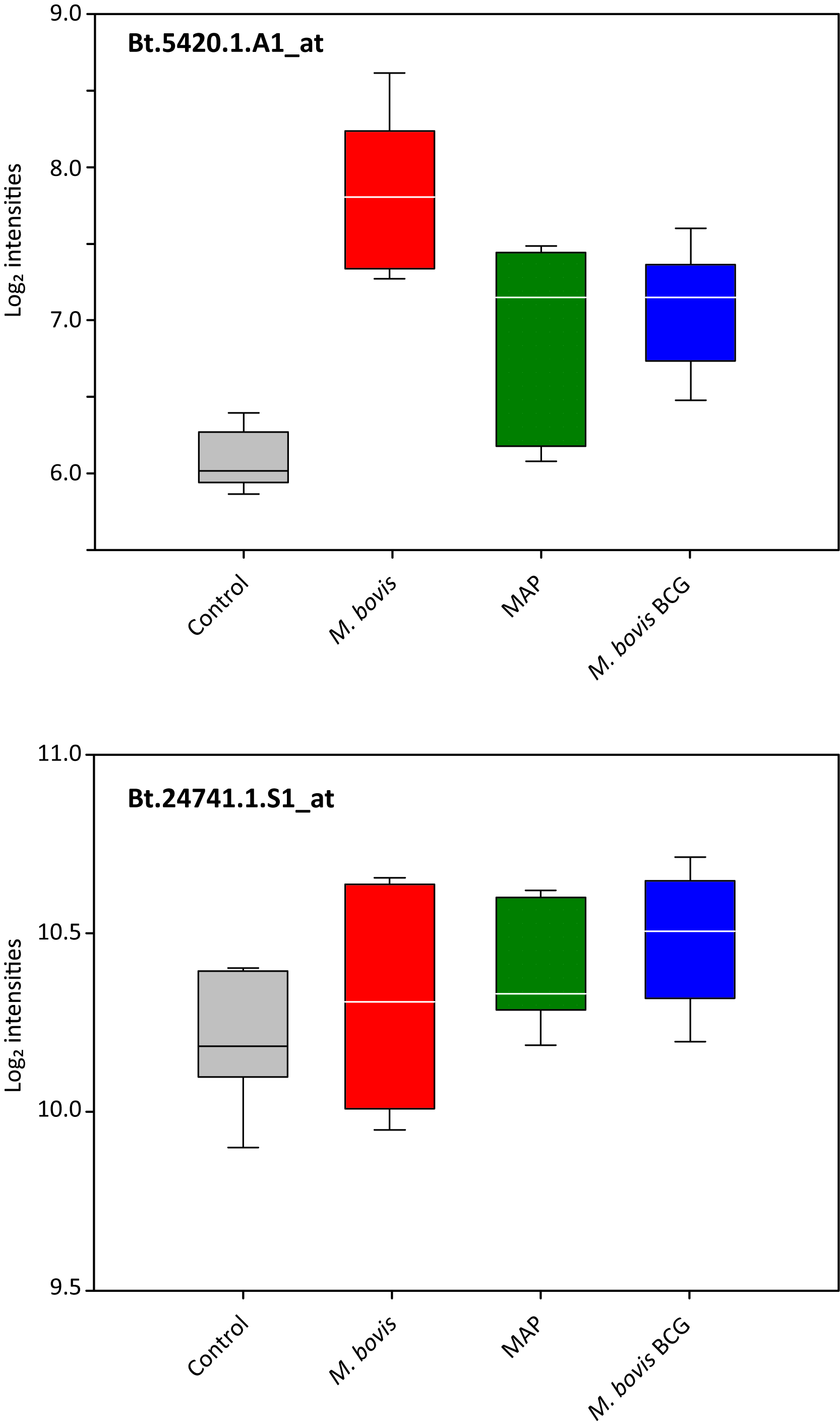

Supplement: Supplementary file 8 [file Image_2.TIF]
